# Congenital toxoplasmosis with severe neurological disease in a referral hospital in Peru

**DOI:** 10.17843/rpmesp.2022.392.10897

**Published:** 2022-06-30

**Authors:** Julio Maquera-Afaray, Medalit Luna-Vilchez, Blanca Salazar-Mesones, Christian Chiara-Chilet, Alexander Cordero-Campos, José W. López

**Affiliations:** 1 Instituto Nacional de Salud del Niño San Borja, Lima, Peru Instituto Nacional de Salud del Niño San Borja Lima Peru; 2 Universidad Privada de Tacna, Tacna, Peru. Universidad Privada de Tacna Universidad Privada de Tacna Tacna Peru; 3 Universidad Peruana Cayetano Heredia, Lima, Peru. Universidad Peruana Cayetano Heredia Universidad Peruana Cayetano Heredia Lima Peru; 4 Universidad Científica del Sur, Lima, Peru. Universidad Científica del Sur Universidad Científica del Sur Lima Peru

**Keywords:** Congenital Toxoplasmosis, Hydrocephalus, Microcephaly, Chorioretinitis, Central Diabetes Insipidus

## Abstract

The aim of this study was to describe the epidemiological, clinical, and therapeutic characteristics of patients diagnosed with congenital toxoplasmosis (CT) with severe neurological disease. We reviewed the medical records of patients under 1 year of age with positive IgM test for *Toxoplasma gondii* and brain, eye, and/or hearing involvement. This study was carried out at the Instituto Nacional de Salud del Niño San Borja (INSNSB), Lima, Peru. Twenty-one patients diagnosed with CT were evaluated; 57.1% were female, and the median age at diagnosis was 3.1 months (IQR: 1.7-7.3). The main central nervous system manifestations were hydrocephalus (76.2%), intracranial calcifications (52.4%), microcephaly (42.9%), and convulsions (25.6%); the most frequent ocular manifestation was chorioretinitis (38.1%). In conclusion, 64% of CT cases had one or more manifestations of severe neurological disease.

## INTRODUCTION

Congenital toxoplasmosis (CT) is a disease caused by *T. gondii*, a protozoan parasite that represents a public health problem still neglected in several countries, including Peru ^1^; being a preventable cause of neurological damage and congenital blindness ^1^ ^,^ ^2^.

More than one third of the world’s population is estimated to be infected by this parasite; however, if infection occurs during pregnancy, it can cause damage to the newborn’s central nervous system ^3^. Worldwide, the estimated incidence of congenital infection is 400 to 4,000 new cases each year ^4^, and the disease burden of CT in disability-adjusted life years (DALYs) is 1.20 million per year ^5^. Some reports indicate that CT cases are more severe in Latin American countries, due to a probable exposure to more virulent strains (type I) ^6^.

In Peru, reports on CT are scarce and isolated ^7^. Therefore, the aim of this study was to describe the epidemiological, clinical and therapeutic characteristics of patients diagnosed with CT with encephalic, ocular and/or auditory involvement in a national pediatric referral institute in Lima, Peru.

**KEY MESSAGES**

Motivation for the study: Congenital toxoplasmosis (CT) is a neglected public health problem in Peru, with scarce clinical and epidemiological information.

Main findings: Of the CT cases, 64% were found to have one or more manifestations of severe neurological disease; 81% were from the provinces. The main manifestations were hydrocephalus (76.2%), intracranial calcifications (52.4%), microcephaly (42.9%), and chorioretinitis (38.1%).

Implications: The study findings reveal the need to improve the registration, surveillance and research on congenital infections in the Peruvian population.

## THE STUDY

### Study site

INSNSB is a pediatric referral hospital center for high complexity surgical and specialized medical management.

### Study design

The study design was observational, descriptive and retrospective.

### Study population and selection criteria

Patients under 1 year of age with serological diagnosis of CT who attended the INSNSB in Lima, Peru, during the period from January 2015 to December 2019 were included. Patients with CT diagnosis were defined as those with the presence of IgM antibodies for *T. gondii* greater than 350 IU/mL, using the enzyme immunoassay technique (ELISA), Euroimmun commercial kit; and according to the CT classification by Desmonts and Couvreur, only children with severe neurological disease were considered ^8^. CT cases without neurological involvement and with incomplete data in the clinical records for the analyzed variables were excluded.

### Study variables

Demographic variables such as age, sex (male, female), place of birth, comorbidities, gestational age and birth weight in grams were registered. Gestational age was categorized; and premature cases were defined as those with gestational age at birth less than 37 weeks. Birth weight was categorized as very low birth weight (< 1,500 g), low birth weight (< 2,500 g), and adequate birth weight (between 2,500 and 3,999 g). Findings related to brain involvement such as intracranial calcifications, hydrocephalus, microcephaly, agenesis/dysgenesis of the corpus callosum and cerebellar hypoplasia were established by transfontanellar ultrasound, computerized tomography and/or brain MRI. Ocular involvement was determined by fundus evaluation performed by an ophthalmologist, who determined the presence of chorioretinitis or other ocular alterations. In addition, auditory and visual evoked potential tests were conducted by the neurophysiology service of the INSNSB, as a complementary study to establish the presence and extent of hypoacusis and decreased visual acuity, respectively. The use of systemic and intraocular antiparasitic treatment was registered, according to the case, as well as the use of adjuvant corticosteroid therapy. 

### Statistical analysis

We used Stata® v16 statistical software (Stata Corporation, College Station, Texas, USA) for descriptive statistics. Quantitative variables were represented by medians and interquartile range (IQR), according to the analysis of data distribution, while qualitative variables were summarized using frequencies and percentages.

### Ethical aspects

The study protocol was approved by the INSNSB Research Ethics Committee, under the institutional code PI-176-2018.

## FINDINGS

**Figure 1:**
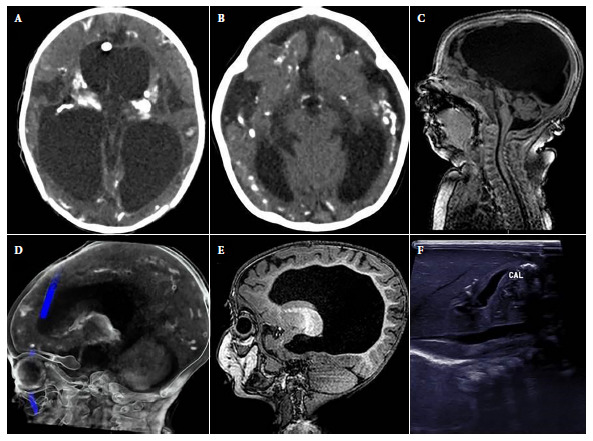
Abnormal findings in images of the cases. A). hydrocephalus and intracranial calcifications in cross-sectional brain computerized tomography. B). Decreased brain parenchymal volume with ventricular dilatation, microcephaly and intracranial calcifications in cross section of brain computerized tomography. C). Cerebellar hypoplasia in sagittal section of brain MRI. D). Intracranial calcifications in sagittal section of cerebral computerized tomography. E). Dysgenesis of the corpus callosum in sagittal section of brain MRI. F). Hepatic calcifications in abdominal ultrasound.

During the study period, 79 patients with positive IgM results for *T. gondii* were identified at INSNSB, of which 33 were patients under 1 year of age (CT cases), 21 (63.6%) of them were included because they showed clinical manifestations of CT with severe neurological disease. The main epidemiological and clinical characteristics are detailed in Table 1, and the images of the most representative brain lesions of the cases are shown in Figure 1.

**Table 1:** Clinical-epidemiological characteristics of patients with CT and severe neurological disease (n=21).

Characteristics	N	%
Female	12	57.1
Age (months) ^a^	3.13	(1.7-7.3)
Prematurity (n=20)	10	50
Birth weight in grams ^a^	2905	(1805-3545)
LBW	8	40.0
VLBW	2	10.0
Manifestations of the central nervous system (n=21)		
Hydrocephaly	16	76.2
Intracranial calcifications	11	52.4
Microcephalia	9	42.9
Convulsions	6	25.6
Dysgenesis of the corpus callosum	4	19.1
Cerebellar hypoplasia	2	9.5
Central diabetes insipidus	2	9.5
Trigonocephaly	1	4.8
Craniosynostosis	1	4.8
Ocular manifestations (n=16)		
Chorioretinitis	8	38.1
Vitritis	4	19.0
Retinal detachment	4	19.0
Cataracts	4	19.0
Posterior uveitis	1	4.8.0
Choroiditis	1	4.8
Exophthalmos	1	4.8
Macular scar	1	4.8
Auditory evoked potentials (n=10)		
Mild sensorineural hearing loss	6	60.0
Profound sensorineural hearing loss	4	40.0
Bilateral	10	100.0
Visual evoked potentials (n=15)		
Visual pathway dysfunction	6	40.0
Absence of visual pathways	9	60.0
Unilateral	3	20.0
Bilateral	12	80.0

a Median (interquartile range).

LBW = low birth weight. VLBW = very low birth weight.

The median age at diagnosis was 3.1 months (IQR: 1.7-7.3), and only 3/21 were diagnosed during the first month of life. None of the mothers were aware of the CT diagnosis or received treatment during gestation. Of the patients, 19.1% were from Lima (4/21), while 81% were from provinces (total 17/21; Ancash (3), Ayacucho (2), Piura (2), Huánuco (2), Amazonas (1), Arequipa (1), Cusco (1), Huancavelica (1), Junín (1), Lambayeque (1), San Martín (1) and Ucayali (1)). The comorbidities found in 10/21 patients were: congenital heart disease (5), spina bifida (2), myelomeningocele (1), esophageal atresia (1) and bronchopulmonary dysplasia (1). In addition, 19.1% (4/21) presented visceromegaly, and the presence of hepatic calcifications was identified in one case by ultrasound (Figure 1).

Laboratory tests showed the following results (median and IQR): hemoglobin: 11.4 g/dL (IQR: 9.8-12.4), platelet count: 293,000 (IQR: 212,000-536,000), eosinophils: 290 (IQR: 120-520), alanine aminotransferase: (ALT) 28 U/L (IQR: 14-44), total bilirubin: 2.095 mg/dL (IQR: 0.2-5). The cytochemical analysis of cerebrospinal fluid (CSF) carried out in 9/21 patients showed the following: mean cell count 30.2 (SD: 19.2) cells/mm^3^ (polymorphonuclear: 21.5, SD: 17.9; mononuclear: 78.5, SD: 17.9), glycorrhachia: 29.3 mg/dL (SD: 17.4), CSF protein: 484 mg/dL (IQR: 109-589), and adenosine deaminase: (ADA) 484 U/L (IQR: 109-589). Antiparasitic treatment was used in 13/21 patients and only three received the combination of pyrimethamine plus sulfadiazine, other details of the treatment are shown in Table 2.

**Table 2:** Characteristics of patients with CT who received antiparasitic treatment (n=13).

N.°	Age/ Sex	Characteristics at birth		Manifestations			Treatment	Adjuvant corticosteroid therapy
		Prematurity	LBW/VLBW	Ocular	Auditory	Cerebral		
1	27 days / F	Yes	LBW	Chorioretinitis VEP: bilateral dysfunction	AEP: NP	No	TMP-SMX + clindamycin	No
2	1 month / F	No	AW	Chorioretinitis VEP: NP	AEP: NP.	Hydrocephalus, intracranial calcifications, convulsions	TMP-SMX	No
3	26 days / M	Yes	LBW	Vitreitis, Retinal detachment VEP: NP	AEP: NP	Hydrocephalus, intracranial calcifications, microcephaly	Pyrimethamine + sulfadiazine	Yes
4	1 month /F	Yes	LBW	Cataracts VEP: NP	AEP: NP	Hydrocephalus, intracranial calcifications	Pyrimethamine + sulfadiazine	No
5	2 months / M	No	AW	EVP: bilateral absence	AEP: Mild bilateral hearing loss	Hydrocephalus, convulsions	Pyrimethamine + sulfadiazine ( TMP-SMX + clindamycin ( TMP-SMX	No
6	4 months /F	No	LBW	Chorioretinitis VEP: bilateral absence	AEP: NP	Hydrocephalus, intracranial calcifications	TMP-SMX + clindamycin	Yes
7	2 months / M	No	AW	Chorioretinitis. VEP: bilateral absence	AEP: Mild bilateral hearing loss	Hydrocephalus, convulsions, diabetes insipidus.	TMP-SMX + clindamycin	Yes
8	8 days / F	Yes	LBW	Vitreitis, posterior uveitis, Retinal detachment VEP: bilateral absence	AEP: Mild bilateral hearing loss	Hydrocephalus, intracranial calcifications, dysgenesis of the corpus callosum	Azithromycin + clindamycin ( TMP-SMX + clindamycin ( TMP-SMX * Intraocular treatment	Yes
9	7 months / F	Yes	VLBW	Chorioretinitis, Retinal detachment VEP: NP	AEP: NP	Hydrocephalus, intracranial calcifications, convulsions	TMP-SMX + azithromycin ( TMP-SMX + clindamycin ( TMP-SMX	Yes
10	2 months / F	No	LBW	Chorioretinitis VEP: unilateral dysfunction	AEP: NP	Intracranial calcifications, microcephaly	TMP-SMX + azithromycin ( TMP-SMX	Yes
11	7 months / F	No	AW	Chorioretinitis. VEP: unilateral dysfunction	AEP: NP	Hydrocephalus, intracranial calcifications, microcephaly	TMP-SMX	Yes
12	5 months / M	ND	ND	Chorioretinitis. VEP: bilateral absence	AEP: Mild bilateral hearing loss	Intracranial calcifications, microcephaly, convulsions.	TMP-SMX	No
13	1 month / F	Yes	LBW	Vitreitis VEP: bilateral absence	AEP: Profound bilateral hearing loss	Hydrocephalus, calcifications, microcephaly, cerebellar hypoplasia, diabetes insipidus.	Pyrimethamine + clindamycin ( TMP-SMX * Intraocular treatment	Yes

AW: adequate weight. LBW: low birth weight. VLBW: very low birth weight. ND: not documented. NP: not performed. AEP: auditory evoked potentials. VEP: visual evoked potentials. TMP-SMX: trimethoprim-sulfamethoxazole.

## DISCUSSION

This study shows a high percentage of CT cases with severe neurological disease (64%), describes the largest number of patients with this diagnosis, and was carried out in a single institution in Peru over a period of five years. However, there are few and isolated published reports of CT with severe neurological disease in Peru ^7^, despite the existence of high seroprevalence of toxoplasmosis in pregnant women, which varies between 32.5% and 94.5% ^9^.

Different routes of transmission of CT have been reported: 1) primary infection during or shortly before pregnancy; 2) reactivation of *T. gondii* in an HIV-infected or otherwise immune-compromised mother, and 3) secondary to infection by an atypical genotype of *T. gondii *in a pregnant woman immune to a typical genotype ^10^. Most CT cases are asymptomatic, the severity of fetal damage by *T. gondii* occurs mainly when the maternal infection occurs early in pregnancy (during the first trimester); however, the permeability of the placental barrier that allows the passage of *T. gondii* to the fetus is lower in the first trimester (equivalent to 10%), while during the second and third trimester permeability reaches 30 and 70%, respectively, in these cases the risk of fetal infection is higher, but with less possibility of causing neurological damage ^11^. 

On the other hand, it has been previously reported that the risk of CT with severe neurological disease, with clinical manifestations included in the classic Sabin and Pinkerton triad (hydrocephalus or microcephaly, intracranial calcifications and chorioretinitis), is higher in South American countries (according to data from Brazil and Colombia) compared to European countries, with a comparative risk in ocular lesions of 47% versus 14%, and for intracranial lesions of 53% versus 9% ^12^. Likewise, Olariu *et al*. identified that 85% of the cases with clinical suspicion and laboratory confirmation of CT showed signs of severe neurological disease in the United States ^13^, while that figure was 64% in our study; it is worth mentioning that the INSNSB is a referral hospital that receives patients with diseases requiring neurosurgical management (such as hydrocephalus). Another series of CT cases in Peru reported 16 cases over a period of 15 years, two patients presented hydrocephalus (12.5%), four had microcephaly (25%) and none showed intracranial calcifications or ocular involvement ^14^. In addition, few cases of central diabetes insipidus secondary to CT have been reported in the literature; in our study we found two patients with this condition involving the hypothalamic-pituitary axis ^15^. Sensorineural hearing loss associated with CT is another clinical manifestation that should be evaluated, its prevalence varies and can reach up to 26% of cases, therefore monitoring and periodic audiometric evaluations are necessary and recommended in those patients diagnosed with CT ^16^.

Treatment for CT includes a prenatal approach, with the purpose of preventing infection of the fetus, and in case of infection, offering early treatment during gestation; the postnatal approach focuses on treating the infection and preventing sequelae ^17^. The treatment of choice for postnatal CT is based on the combination of pyrimethamine and sulfadiazine for a period of 12 months, both drugs have antiparasitic action against the tachyzoite forms of *T. gondii*; however, these drugs have not shown effectiveness in eliminating the cystic forms (bradyzoite) of the parasite, especially in the central nervous system and ocular system ^17^ ^,^ ^18^. The use of other drugs, alone or in combination with pyrimethamine, such as sulfadoxine, clindamycin, cotrimoxazole, atovaquone and azithromycin, have also been described as treatment alternatives ^17^, due to the lack of availability and access to pyrimethamine and sulfadiazine. Adjuvant corticosteroid therapy is used in cases with active ocular involvement, such as chorioretinitis, and in cases with CSF proteins greater than 1 g/dL ^19^.

When it comes to prevention, it is important to strengthen public education, especially for pregnant women; information must be detailed and practical in order to allow clear communication between the physician and the patient, with the purpose of improving adherence to measures and habits, fundamentally hygiene, which reduces exposure to *T. gondii* ^1^ ^,^ ^4^. In addition, countries such as France have an effective gestational screening and early treatment program that has been shown to reduce the prevalence and severity of CT; however, other countries with sociodemographic characteristics similar to ours, such as Morocco and Colombia, do screen pregnant women for CT, but have shown difficulties in the follow-up and treatment of diagnosed cases ^20^. 

A limitation of the study was that the cases were selected from only one institution; and therefore, the results may not reflect the real situation of CT in the country. Likewise, only patients with severe neurological disease and with postnatal diagnosis were included, because INSNSB, being a reference health facility, is focused on the management of surgical pathologies in pediatric patients and does not provide care to pregnant women.

In conclusion, our study reports a high percentage of CT cases with severe neurological disease mainly hydrocephalus, intracranial calcifications, microcephaly, and chorioretinitis. Most cases were diagnosed after one month of age and received alternative treatment. It is important to mention that in Peru congenital CT and other congenital infections are underreported and can therefore be considered as neglected. Therefore, national surveillance and research are required in order to identify the real implications and repercussions of CT in the Peruvian population, allowing the development of guidelines with appropriate strategies for prevention, early diagnosis and timely treatment during gestation and for the newborn, with access and availability of diagnostic methods and medications of choice.

## References

[1] Singh S (2016). Congenital toxoplasmosis Clinical features, outcomes, treatment, and 
prevention. Trop Parasitol.

[2] Alvarado-Socarras JL, Meneses-Silvera K, Zarate-Vergara AC, Guerrero-Gómez C, Rodríguez-Morales AJ (2017). No todo es zika: toxoplasmosis congénita, ¿aún prevalente en Colombia?. Rev Peru Med Exp Salud Publica.

[3] Moncada PA, Montoya JG (2012). Toxoplasmosis in the fetus and newborn: an update on prevalence, diagnosis and treatment. Expert Rev Anti Infect Ther.

[4] Kaye A (2011). Toxoplasmosis Diagnosis, Treatment, and Prevention in Congenitally Exposed 
Infants. J Pediatr Health Care.

[5] Torgerson PR, Mastroiacovo P (2013). The global burden of congenital toxoplasmosis: a systematic review. Bull World Health Organ.

[6] Gómez JE (2011). First Colombian multicentric newborn screening for congenital toxoplasmosis. PLoS Negl Trop Dis.

[7] Samalvides SK, Milla LM, Vila JR, Espinoza IO, Guillén-Pinto D (2014). Tres formas clínico-
radiológicas de compromiso neurológico por toxoplasmosis congénita. Rev Neuropsiquiatr.

[8] Hayde M, Pollak A, Ambroise- Thomas P, Petersen E (2000). Congenital toxoplasmosis. Scientific background, clinical 
management and control.

[9] Reátegui BC, Vela GL (2011). Factores socioeconómicos - epidemiológicos y su relación con la 
seroprevalencia de toxoplasmosis en gestantes atendidas en los hospitales "Felipe 
Arriola" y "César Garayar", Iquitos, Perú, 2009. Neotropical Helminthology.

[10] Lindsay DS, Dubey JP (2011). Toxoplasma gondii the changing paradigm of congenital 
toxoplasmosis. Parasitology.

[11] Robert-Gangneux F, Dardé ML (2012). Epidemiology of and diagnostic strategies for 
toxoplasmosis. Clin Microbiol Rev.

[12] Thiébaut R, Leproust S, Chêne G, Gilbert R, SYROCOT (Systematic Review on Congenital Toxoplasmosis) study group (2007). Effectiveness of prenatal treatment for congenital 
toxoplasmosis a meta-analysis of individual patients' data. Lancet.

[13] Olariu TR, Remington JS, McLeod R, Alam A, Montoya JG (2011). Severe congenital 
toxoplasmosis in the United States clinical and serologic findings in untreated infants.. Pediatr Infect Dis J.

[14] Martinez Ortiz P, Ticona Vildoso M (2012). Toxoplasmosis congénita en el hospital regional 
Honorio Delgado Espinoza de Arequipa, 1996 a 2011. Revista Médica Basadrina.

[15] Mohamed S, Osman A, Al Jurayyan NA, Al Nemri A, Salih MA (2014). Congenital toxoplasmosis 
presenting as central diabetes insipidus in an infant a case report. BMC Res Notes..

[16] Brown ED, Chau JK, Atashband S, Westerberg BD, Kozak FK (2009). A systematic review of 
neonatal toxoplasmosis exposure and sensorineural hearing loss. Int J Pediatr 
Otorhinolaryngol.

[17] Petersen E, Schmidt DR (2003). Sulfadiazine and pyrimethamine in the postnatal treatment 
of congenital toxoplasmosis what are the options?. Expert Rev Anti Infect Ther.

[18] Serranti D, Buonsenso D, Valentini P (2011). Congenital toxoplasmosis treatment. Eur Rev 
Med Pharmacol Sci.

[19] Baquero-Artigao F, del Castillo-Martín F, Fuentes-Corripio I, Goncé-Mellgren A, Fortuny-Guasch C, de la Calle Fernández-Miranda M (2013). Guía de la Sociedad Española de Infectología Pediátrica para el diagnóstico y tratamiento de la toxoplasmosis congénita. An Pediatr.

[20] El Bissati K, Levigne P, Lykins J, Adlaoui EB, Barkat A, Berraho A (2018). Global initiative for congenital toxoplasmosis an observational and international comparative clinical analysis. Emerg Microbes Infect.

