# Supplementary material for: Congenital toxoplasmosis with severe neurological disease in a referral hospital in Peru
Source: Rev Peru Med Exp Salud Publica. 2022 Jun 30;39(2):208–13. doi: 10.17843/rpmesp.2022.392.10897 (PMC11397690; doi:10.17843/rpmesp.2022.392.10897)
Supplement: Translation

# Translation: Toxoplasmosis congénita con enfermedad neurológica severa en un hospital de referencia del Perú

**Authors:** Julio Maquera-Afaray, Medalit Luna-Vilchez, Blanca Salazar-Mesones, Christian Chiara-Chilet, Alexander Cordero-Campos, José W. López

**Affiliations:** 1 Instituto Nacional de Salud del Niño San Borja, Lima, Perú.; 2 Universidad Privada de Tacna, Tacna, Perú.; 3 Universidad Peruana Cayetano Heredia, Lima, Perú.; 4 Universidad Científica del Sur, Lima, Perú.

**Keywords:** Toxoplasmosis Congénita, Hidrocefalia, Microcefalia, Coriorretinitis, Diabetes Insípida Central

## Abstract

El propósito del presente estudio fue describir las características epidemiológicas, clínicas, y terapéuticas de pacientes con diagnóstico de toxoplasmosis congénita (TC) con enfermedad neurológica severa. Se revisaron las historias clínicas de los pacientes menores de 1 año con serología IgM positiva para *Toxoplasma gondii* y compromiso encefálico, ocular y/o auditivo. El estudio se realizó en el Instituto Nacional de Salud del Niño San Borja (INSN-SB) en Lima, Perú. Se evaluaron a 21 pacientes con diagnóstico de TC, el 57,1% fueron del sexo femenino y la mediana de edad al momento del diagnóstico fue 3,1 meses (RIC: 1,7-7,3). Las principales manifestaciones del sistema nervioso central fueron hidrocefalia (76,2%), calcificaciones intracraneales (52,4%), microcefalia (42,9%), y convulsiones (25,6%); la manifestación ocular más frecuente fue la coriorretinitis (38,1%). En conclusión, 64% de los casos de TC tuvieron una o más manifestaciones de enfermedad neurológica severa.

## INTRODUCCIÓN

La toxoplasmosis congénita (TC) es una enfermedad causada por *T. gondii*, un parásito protozoo que constituye un problema de salud pública aún desatendido en diversos países, incluyendo al Perú ^1^; siendo una causa prevenible de daño neurológico y ceguera congénita ^1^ ^,^ ^2^.

Se estima que más de un tercio de la población mundial está infectada por este parásito; no obstante, si la infección ocurre durante el embarazo puede provocar daño en el sistema nervioso central del recién nacido ^3^. A nivel mundial, la incidencia estimada de la infección congénita es de 400 a 4000 nuevos casos cada año ^4^, y la carga de la enfermedad por TC establecida en años de vida ajustados a discapacidad (AVAD) es de 1,20 millones por año ^5^. Algunos reportes indican que los casos de TC son más severos en países de Latinoamérica, debido a una probable exposición a cepas más virulentas (tipo I) ^6^. 

En Perú, los reportes sobre TC son escasos y aislados ^7^. Por tanto, el objetivo del presente estudio fue describir las características epidemiológicas, clínicas y terapéuticas en pacientes con diagnóstico de TC con compromiso encefálico, ocular y/o auditivo en un instituto pediátrico de referencia nacional en Lima, Perú.

**MENSAJE CLAVE**

Motivación para realizar el estudio: .la toxoplasmosis congénita (TC) es un problema de salud pública desatendido, con escasa información clínica y epidemiológica.

Principales hallazgos: se encontró que el 64% de los casos de TC tuvieron una o más manifestaciones de enfermedad neurológica severa. El 81% eran procedentes de una ciudad de provincia. Las principales manifestaciones fueron hidrocefalia (76,2%), calcificaciones intracraneales (52,4%), microcefalia (42,9%), y coriorretinitis (38,1%).

Implicancias: los hallazgos del estudio revelan la necesidad de mejorar el registro, vigilancia e investigación sobre las infecciones congénitas en la población peruana.

## EL ESTUDIO

### Lugar de estudio

El INSN-SB es un centro hospitalario pediátrico de referencia para manejo quirúrgico y especializado de alta complejidad. 

### Diseño del estudio

El diseño del estudio fue de tipo observacional, descriptivo, retrospectivo.

### Población de estudio y criterios de selección

Se incluyeron pacientes menores de 1 año con diagnóstico serológico de TC atendidos en el INSN-SB de Lima, Perú, durante el período comprendido entre enero del 2015 a diciembre del 2019. Se definió como paciente con diagnóstico de TC aquellos con presencia de anticuerpos IgM para *T. gondii* con un valor mayor de 350 UI/mL, mediante la técnica de inmunoensayo enzimático (ELISA), kit comercial Euroimmun; y de acuerdo con la clasificación de TC por Desmonts and Couvreur se consideraron solo a los niños con enfermedad neurológica severa ^8^. Se excluyeron los casos de TC sin compromiso neurológico y con datos incompletos en la historia clínica para las variables analizadas. 

### Variables de estudio

Se registraron variables demográficas como edad, sexo (masculino, femenino), lugar de nacimiento, comorbilidad, edad gestacional y peso al nacimiento en gramos La edad gestacional se categorizó y definió como prematuridad aquellos con edad gestacional al nacimiento menor de 37 semanas. El peso al nacimiento se categorizó en muy bajo peso al nacer (< 1500 g), bajo peso al nacer (< 2500 g), y adecuado peso (entre 2500 y 3999 g). Los hallazgos correspondientes al compromiso cerebral como calcificaciones intracraneales, hidrocefalia, microcefalia, agenesia/disgenesia del cuerpo calloso e hipoplasia cerebelosa fueron establecidos mediante ecografía transfontanelar, tomografía y/o resonancia magnética cerebral. Mientras que el compromiso ocular fue determinado mediante la evaluación del fondo de ojo realizada por un médico oftalmólogo, quien determinó la presencia de coriorretinitis u otra alteración ocular. Además, se realizaron potenciales evocados auditivos y visuales por el servicio de neurofisiología del INSN-SB, como estudio complementario para establecer la presencia y grado de hipoacusia y disminución de la agudeza visual, respectivamente. Se registró el tratamiento antiparasitario sistémico e intraocular, de acuerdo con el caso, así como el uso de corticoterapia adyuvante. 

### Análisis estadístico

Se utilizó el *software* estadístico Stata® v16 (Stata Corporation, College Station, Texas, USA) para la estadística descriptiva. Las variables cuantitativas fueron representadas por medianas y rango intercuartílico (RIC), de acuerdo con el análisis de la distribución de los datos, mientras que las variables cualitativas se resumen usando frecuencias y porcentajes. 

### Aspectos éticos

El protocolo de estudio fue aprobado por el Comité de Ética en Investigación del INSN-SB, bajo el código institucional PI-176-2018.

## HALLAZGOS

Durante el periodo de estudio se identificaron 79 pacientes con serología IgM positiva para *T. gondii* en el INSN-SB, de los cuales 33 fueron pacientes menores de 1 año (casos de TC), 21 (63,6%) de ellos fueron incluidos por presentar manifestaciones clínicas de TC con enfermedad neurológica severa. Las principales características epidemiológicas y clínicas se detallan en la Tabla 1, y las imágenes de las lesiones cerebrales más representativas de los casos se muestran en la Figura 1.

**Tabla 1:** Características clínico-epidemiológicas de los pacientes con TC y enfermedad neurológica severa (n=21).

Características	N	%
Sexo Femenino	12	57,1
Edad (meses)^a^	3,13	(1,7-7,3)
Prematuridad (n=20)	10	50,0
Peso al nacimiento en gramos^a^	2905	(1805-3545)
BPN	8	40,0
MBPN	2	10,0
Manifestaciones del sistema nervioso central (n=21)		
Hidrocefalia	16	76,2
Calcificaciones intracraneales	11	52,4
Microcefalia	9	42,9
Convulsiones	6	25,6
Disgenesia del cuerpo calloso	4	19,1
Hipoplasia cerebelosa	2	9,5
Diabetes insípida central	2	9,5
Trigonocefalia	1	4,8
Craneosinostosis	1	4,8
Manifestaciones oculares (n=16)		
Coriorretinitis	8	38,1
Vitreitis	4	19,0
Desprendimiento de retina	4	19,0
Catarata	4	19,0
Uveítis posterior	1	4,8
Coroiditis	1	4,8
Exoftalmos	1	4,8
Cicatriz macular	1	4,8
Potenciales evocados auditivos (n=10)		
Hipoacusia neurosensorial leve	6	60,0
Hipoacusia neurosensorial profunda	4	40,0
Bilateral	10	100,0
Potenciales evocados visuales (n=15)		
Disfunción de las vías visuales	6	40,0
Ausencia de las vías visuales	9	60,0
Unilateral	3	20,0
Bilateral	12	80,0

a Mediana (rango intercuartílico)

BPN: bajo peso al nacer. MBPN: muy bajo peso al nacer.

**Figura 1:** Hallazgos anormales en imágenes de los casos. A). hidrocefalia y calcificaciones intracraneales en corte transversal de tomografía cerebral. b). disminución del volumen del parénquima cerebral con dilatación ventricular, microcefalia y calcificaciones intracraneales en corte transversal de tomografía cerebral. c). hipoplasia cerebelosa en corte sagital de RMN cerebral. D). calcificaciones intracraneales en corte sagital de tomografía cerebral. E). disgenesia del cuerpo calloso en corte sagital de RMN cerebral. F). calcificaciones hepáticas en ecografía abdominal.

La mediana de edad al momento del diagnóstico de los pacientes fue 3,1 meses (RIC: 1,7-7,3), y solo 3/21 fueron diagnosticados durante el primer mes de vida. Ninguna madre conocía el diagnóstico de TC ni recibió tratamiento durante la gestación. El 19,1% eran procedentes de Lima (4/21), mientras que 81% de provincia (total 17/21; Ancash (3), Ayacucho (2), Piura (2), Huánuco (2), Amazonas (1), Arequipa (1), Cusco (1), Huancavelica (1), Junín (1), Lambayeque (1), San Martín (1) y Ucayali (1)). Las comorbilidades registradas en 10/21 pacientes fueron cardiopatía congénita (5), espina bífida (2), mielomeningocele (1), atresia esofágica (1) y displasia broncopulmonar (1). Además, 19,1% (4/21) presentó visceromegalia y se identificó presencia de calcificaciones hepáticas en un caso mediante estudio ecográfico (Figura 1).

Los análisis de laboratorio mostraron una mediana de hemoglobina de 11,4 g/dL (RIC: 9,8-12,4), recuento de plaquetas de 293000 (RIC: 212000-536000), eosinófilos 290 (RIC: 120-520), alanina-aminotransferasa (ALT) 28 U/L (RIC: 14-44), bilirrubina total 2,095 mg/dL (RIC: 0,2-5). El análisis citoquímico del líquido cefalorraquídeo (LCR) fue realizado en 9/21 pacientes y se encontró como media del recuento celular 30,2 (DE: 19,2) células/mm3 (polimorfonucleares 21,5, DE: 17,9; mononucleares 78,5, DE: 17,9), glucorraquia 29,3 mg/dL (DE: 17,4), proteinorraquia 484 mg/dL (RIC: 109-589), y adenosina deaminasa (ADA) 15 U/L (RIC: 11-75). El tratamiento antiparasitario fue empleado en 13/21 pacientes y solo tres recibieron la combinación de pirimetamina más sulfadiazina, otros detalles de los regímenes de tratamiento que recibieron se muestran en la Tabla 2.

**Tabla 2:** Características de los pacientes con TC que recibieron tratamiento antiparasitario (n=13).

N.°	Edad/ Sexo	Características al nacimiento		Manifestaciones			Régimen terapéutico	Corticoterapia adyuvante
		Prematuridad	Peso	Oculares	Auditivas	Cerebrales				
1	27 días / F	Sí	BPN	Coriorretinitis PEV: disfunción bilateral	PEA: NR	No	TMP-SMX + clindamicina	No
2	1 mes / F	No	AP	Coriorretinitis PEV: NR	PEA: NR.	Hidrocefalia, calcificaciones intracraneales, convulsiones	TMP-SMX	No
3	26 días / M	Sí	BPN	Vitreitis, desprendimiento de retina PEV: NR	PEA: NR	Hidrocefalia, calcificaciones intracraneales, microcefalia	Pirimetamina + sulfadiazina	Sí
4	1 mes /F	Sí	BPN	Catarata PEV: NR	PEA: NR	Hidrocefalia, calcificaciones intracraneales	Pirimetamina + sulfadiazina	No
5	2 meses / M	No	AP	PEV: ausencia bilateral	PEA: Hipoacusia bilateral leve	Hidrocefalia, convulsiones	Pirimetamina + sulfadiazina à TMP-SMX + clindamicina à TMP-SMX	No
6	4 meses /F	No	BPN	Coriorretinitis PEV: ausencia bilateral	PEA: NR	Hidrocefalia, calcificaciones intracraneales	TMP-SMX + clindamicina	Sí
7	2 meses / M	No	AP	Coriorretinitis. PEV: ausencia bilateral	PEA: Hipoacusia bilateral leve	Hidrocefalia, convulsiones, diabetes insípida	TMP-SMX + clindamicina	Sí
8	8 días / F	Sí	BPN	Vitreitis, uveítis posterior, desprendimiento de retina PEV: ausencia bilateral	PEA: Hipoacusia bilateral leve	Hidrocefalia, calcificaciones intracraneales, disgenesia del cuerpo calloso	Azitromicina + clindamicina à TMP-SMX + clindamicina à TMP-SMX * Tratamiento intraocular	Sí
9	7 meses / F	Sí	MBPN	Coriorretinitis, desprendimiento de retina PEV: NR	PEA: NR	Hidrocefalia, calcificaciones intracraneales, convulsiones	TMP-SMX + azitromicina à TMP-SMX + clindamicina à TMP-SMX	Sí
10	2 meses / F	No	BPN	Coriorretinitis PEV: disfunción unilateral	PEA: NR	Calcificaciones intracraneales, microcefalia	TMP-SMX + azitromicina à TMP-SMX	Sí
11	7 meses / F	No	AP	Coriorretinitis. PEV: disfunción unilateral	PEA: NR	Hidrocefalia, calcificaciones intracraneales, microcefalia	TMP-SMX	Sí
12	5 meses / M	ND	ND	Coriorretinitis. PEV: ausencia bilateral	PEA: Hipoacusia bilateral leve	Calcificaciones intracraneales, microcefalia, convulsiones	TMP-SMX	No
13	1 mes / F	Sí	BPN	Vitreitis PEV: ausencia bilateral	PEA: Hipoacusia bilateral profunda	Hidrocefalia, calcificaciones, microcefalia, hipoplasia cerebelosa, diabetes insípida	Pirimetamina + clindamicina à TMP-SMX * Tratamiento intraocular	Sí

AP: adecuado peso. BPN: bajo peso al nacer. MBPN: muy bajo peso al nacer. ND: no documentado. NR: no realizado. PEA: potenciales evocados auditivos. PEV: potenciales evocados visuales. TMP-SMX: trimetoprim-sulfametoxazol.

## DISCUSIÓN

El presente estudio muestra un alto porcentaje de casos de TC con enfermedad neurológica severa (64%) y describe el mayor número de pacientes con este diagnóstico, realizado en una sola institución de Perú y durante un periodo de cinco años. No obstante, son escasos y aislados los reportes publicados de TC con enfermedad neurológica severa en Perú ^7^, a pesar de existir una alta seroprevalencia de toxoplasmosis en gestantes, que varía entre 32,5 y 94,5% ^9^.

Se han reconocido diferentes vías de transmisión de TC: 1) infección primaria durante o poco antes del embarazo; 2) reactivación de *T. gondii* en una madre infectada por VIH u otro tipo de alteración inmune, y 3) secundaria a la infección por un genotipo atípico de *T. gondii* en una gestante inmune a un genotipo típico ^10^. La mayoría de casos de TC son asintomáticos, la severidad del daño fetal por *T. gondii* ocurre principalmente cuando la infección materna se da en etapas tempranas del embarazo (durante el primer trimestre); sin embargo, la permeabilidad de la barrera placentaria que permite el pasaje parasitario de *T. gondii* al feto, es menor en el primer trimestre (equivalente al 10%), mientras que durante el segundo y tercer trimestre la permeabilidad alcanza un 30 y 70%, respectivamente, siendo en estos casos mayor el riesgo de infección fetal, pero con menos posibilidad de provocar daño neurológico ^11^.

Por otro lado, se ha reportado que el riesgo de TC con enfermedad neurológica severa, con manifestaciones clínicas incluidas en la clásica triada de Sabin y Pinkerton (hidrocefalia o microcefalia, calcificaciones intracraneales y coriorretinitis), es mayor en países de Sudamérica (según datos de Brasil y Colombia) comparado con países de Europa, siendo el riesgo comparativo en lesiones oculares de 47% versus 14%, y lesiones intracraneales de 53% versus 9% ^12^. Asimismo, Olariu *et al*. identificaron que 85% de los casos con sospecha clínica y confirmación laboratorial de TC presentaron manifestaciones de enfermedad neurológica severa en Estados Unidos ^13^, mientras que en nuestro estudio fue de 64%; cabe mencionar que el INSN-SB es un hospital de referencia que recibe pacientes con enfermedades que requieren manejo neuroquirúrgico (como hidrocefalia). En otra serie de casos de TC en Perú, reportó 16 casos durante un periodo de 15 años, dos pacientes presentaron hidrocefalia (12,5%), cuatro con microcefalia (25%) y en ninguno se reportó calcificaciones intracraneales ni compromiso ocular ^14^. Además, en la literatura se han reportado pocos casos de diabetes insípida central secundaria a TC, en nuestro estudio se encontró dos pacientes con esta entidad clínica que compromete el eje hipotálamo-pituitario ^15^. La pérdida auditiva neurosensorial asociada a TC es otra manifestación clínica que debe ser evaluada, su prevalencia es variable y puede alcanzar hasta 26% de los casos, siendo recomendado el monitoreo y evaluaciones audiométricas periódicas en aquellos pacientes con diagnóstico de TC ^16^.

Los regímenes de tratamiento para TC incluyen un abordaje prenatal, con el propósito de prevenir la infección del feto y en caso de la infección ofrecer un tratamiento temprano durante la gestación, mientras que el abordaje posnatal se enfoca en tratar la infección y prevenir las secuelas ^17^. El tratamiento de elección para la TC posnatal se basa en la combinación de pirimetamina y sulfadiazina durante un periodo de 12 meses, ambos medicamentos poseen acción antiparasitaria contra las formas de taquizoito de *T. gondii*; no obstante, estos medicamentos no han mostrado efectividad para eliminar las formas quísticas (bradizoito) del parásito, especialmente del sistema nervioso central y ocular ^17^ ^,^ ^18^. Como alternativas al tratamiento también se han descrito el uso de otros medicamentos usados solos o en combinación con pirimetamina, como son la sulfadoxina, clindamicina, cotrimoxazol, atovaquone y azitromicina ^17^, debido a la falta de disponibilidad y acceso de pirimetamina y sulfadiazina. La corticoterapia adyuvante se emplea en los casos con compromiso ocular activo, como coriorretinitis, y en casos con LCR patológico con proteinorraquia mayor de 1 g/dL ^19^.

En prevención es importante fortalecer la educación pública, sobre todo en gestantes, la información debe ser detallada y práctica para permitir una clara comunicación entre el médico y el paciente, con la finalidad de mejorar la adhesión a las medidas y hábitos, fundamentalmente de higiene, que reducen la exposición a *T. gondii* ^1^ ^,^ ^4^. Además, países como Francia cuenta con un programa de tamizaje gestacional y tratamiento temprano efectivo ha demostrado reducir la prevalencia y severidad de la TC; sin embargo, otros países con características sociodemográficas similares a la nuestra, como Marruecos y Colombia, que realizan el tamizaje de TC en gestantes han evidenciado dificultades en el seguimiento y tratamiento de los casos diagnosticados ^20^. 

El estudio tuvo limitación en la selección de casos que fueron recolectados en una sola institución, por lo cual, no reflejan la casuística real de la TC en el país. Asimismo, se incluyó solo pacientes con enfermedad neurológica severa y con diagnóstico realizado posnatal, debido que INSN-SB, al ser un establecimiento de salud de referencia, está enfocado en manejo de patologías quirúrgicas en pacientes pediátricos y no brinda atención a gestantes.

En conclusión, nuestro estudio demostró un alto porcentaje de casos de TC con enfermedad neurológica severa principalmente hidrocefalia, calcificaciones intracraneales, microcefalia, y coriorretinitis. La mayoría fue diagnosticada después del mes de vida y recibieron regímenes de tratamiento alternativo. Es importante mencionar que en el Perú, existe un subregistro de TC y otras infecciones congénitas, por lo que puede considerarse desatendida; por lo cual, se requiere de vigilancia e investigación a nivel nacional, que visualice las reales implicancias y repercusiones de la TC en la población peruana, que permita elaborar guías con estrategias apropiadas en prevención, diagnóstico temprano y tratamiento oportuno durante la gestación y también en el recién nacido, con acceso y disponibilidad a métodos diagnósticos y medicamentos de elección.
